# An antimicrobial microneedle patch promotes functional healing of infected wounds through controlled release of adipose tissue-derived apoptotic vesicles

**DOI:** 10.1186/s12951-024-02845-2

**Published:** 2024-09-20

**Authors:** Yue Ma, Jia Dong, Maojiao Li, Xinya Du, Zhengbin Yan, Weidong Tian

**Affiliations:** 1 Department of Stomatology, People’s Hospital of Longhua Shenzhen, Shenzhen, Guangdong China; 2 State Key Laboratory of Oral Disease & National Clinical Research Center for Oral Diseases & National Engineering Laboratory for Oral Regenerative Medicine, West China School of Stomatology, Sichuan University, Chengdu, Sichuan China

**Keywords:** Microneedle patch, Antibacterial, Apoptotic vesicles, Infected wound, Adipose tissue

## Abstract

The high incidence and mortality rates associated with acute and chronic wound infections impose a significant burden on global healthcare systems. In terms of the management of wound infection, the reconstruction and regeneration of skin appendages are essential for the recovery of mechanical strength and physiological function in the regenerated skin tissue. Novel therapeutic approaches are a requisite for enhancing the healing of infected wounds and promoting the regeneration of skin appendages. Herein, a novel antimicrobial microneedle patch has been fabricated for the transdermal controlled delivery of adipose tissue-derived apoptotic vesicles (ApoEVs-AT@MNP) for the treatment of infected wounds, which is expected to achieve high-quality scarless healing of the wound skin while inhibiting the bacteria in the infected wound. The microneedle patch (MNP) system possesses adequate mechanical strength to penetrate the skin, allowing the tips to remain inside tissue for continuous active release of biomolecules, and subsequently degrades safely within the host body. In vivo transplantation demonstrates that ApoEVs-AT@MNP not only inhibits bacterial proliferation in infected wounds but also significantly promotes effective and rapid scarless wound healing. Particularly noteworthy is the ability of ApoEVs-AT@MNP to promote the rapid formation of mature, evenly arranged hair follicles in infected wounds, observed as early as 8 days following implantation, which is essential for the restoration of skin function. This rapid development of skin appendages has not been reported this early in previous studies. Therefore, ApoEVs-AT@MNP has emerged as an excellent, painless, non-invasive, and highly promising treatment for infected wounds.

**Supplementary Information:**

The online version contains supplementary material available at 10.1186/s12951-024-02845-2.

## Graphical Abstract

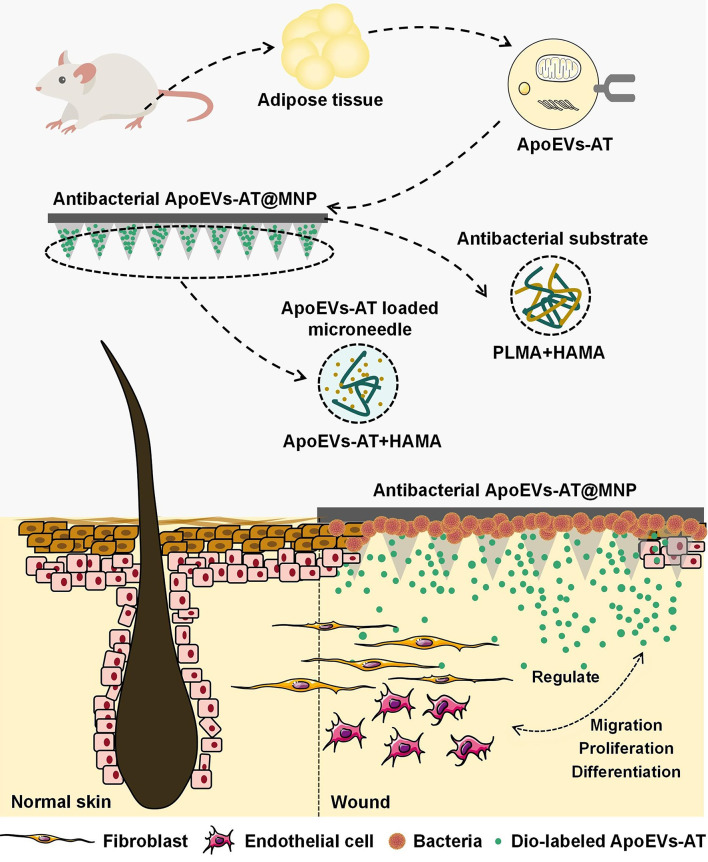

## Introduction

The widespread occurrence and high mortality rates associated with acute and chronic wound infections pose significant challenges for healthcare systems globally, especially in developing regions [1]. Despite the standardization of treatment strategies for infected wounds, significant challenges persist in diagnosing and managing these infections. Issues such as biofilm formation, delayed healing, and drug resistance continue to complicate effective treatment [1, 2]. It is important to recognize that underlying systemic diseases can complicate the healing of infected wounds and exacerbate scar formation. Consequently, there is a critical demand to develop innovative therapeutic strategies to improve the healing process of infected wounds.

Adipose tissue transplantation shows substantial promise for wound treatment [3]. Research indicates that transplanted cells from virtually all kinds of tissues undergo rapid apoptosis, a process vital for regeneration [4–6]. During apoptosis, cells form apoptotic vesicles, which are produced more efficiently than exosomes generated by living cells. Apoptotic vesicles contain proteins, lipids, and nucleic acids, similar to exosomes, but lack the protective membranes that exosomes possess [7]. Apoptotic vesicles offer several advantages: they are readily available, simple to extract, have low immunogenicity, and pose a low tumor risk [5, 8–10]. Their nanoscale dimensions render them ideal for minimally invasive drug delivery. In our previous study, apoptotic vesicles were successfully extracted from adipose tissue (ApoEVs-AT) using gradient centrifugation. The ability of ApoEVs-AT to enhance skin wound regeneration was also preliminarily confirmed in our previous study [11]. However, the newly formed skin lacks regularly arranged and mature skin appendages, which are essential for the recovery of mechanical strength and physiological function of the regenerated skin tissue. The administration of ApoEVs-AT to the lesion site has predominantly relied on the conventional methods of multiple-point and multiple-time injections [11], which is complex, poorly tolerated by patients, and necessitates skilled personnel [12]. Moreover, puncture wounds from injections can easily cause secondary trauma and infection [13]. Furthermore, bacterial biofilms in the wound can unpredictably affect the efficacy of the vesicles. Therefore, there is an urgent need to develop a painless, non-invasive drug delivery system for administering ApoEVs-AT that also possesses antibacterial properties and the ability to stimulate the reconstruction of skin appendages.

MNP has been used extensively for the transdermal delivery of therapeutic agents and have found utilization in many applications including wound healing, myocardial repair, and tumor therapy [14–16]. They can traverse the stratum corneum of the skin without contacting the nociceptive nerve, creating a drug delivery pathway on the skin surface that enables targeted drug penetration to a specified depth within the skin and absorption into the subcutaneous capillary network, thereby facilitating pain-free and non-damaging drug administration [17]. Typically, therapeutic substances are encapsulated within the microneedle arrays of MNP. These patches can furnish a sustained release of the therapeutics over an extended period while minimizing pain and tissue damage [18]. The effectiveness of microneedle mechanical stimulation in promoting the formation of hair follicles and skin appendages has also been validated [19].

**Fig. 1:**
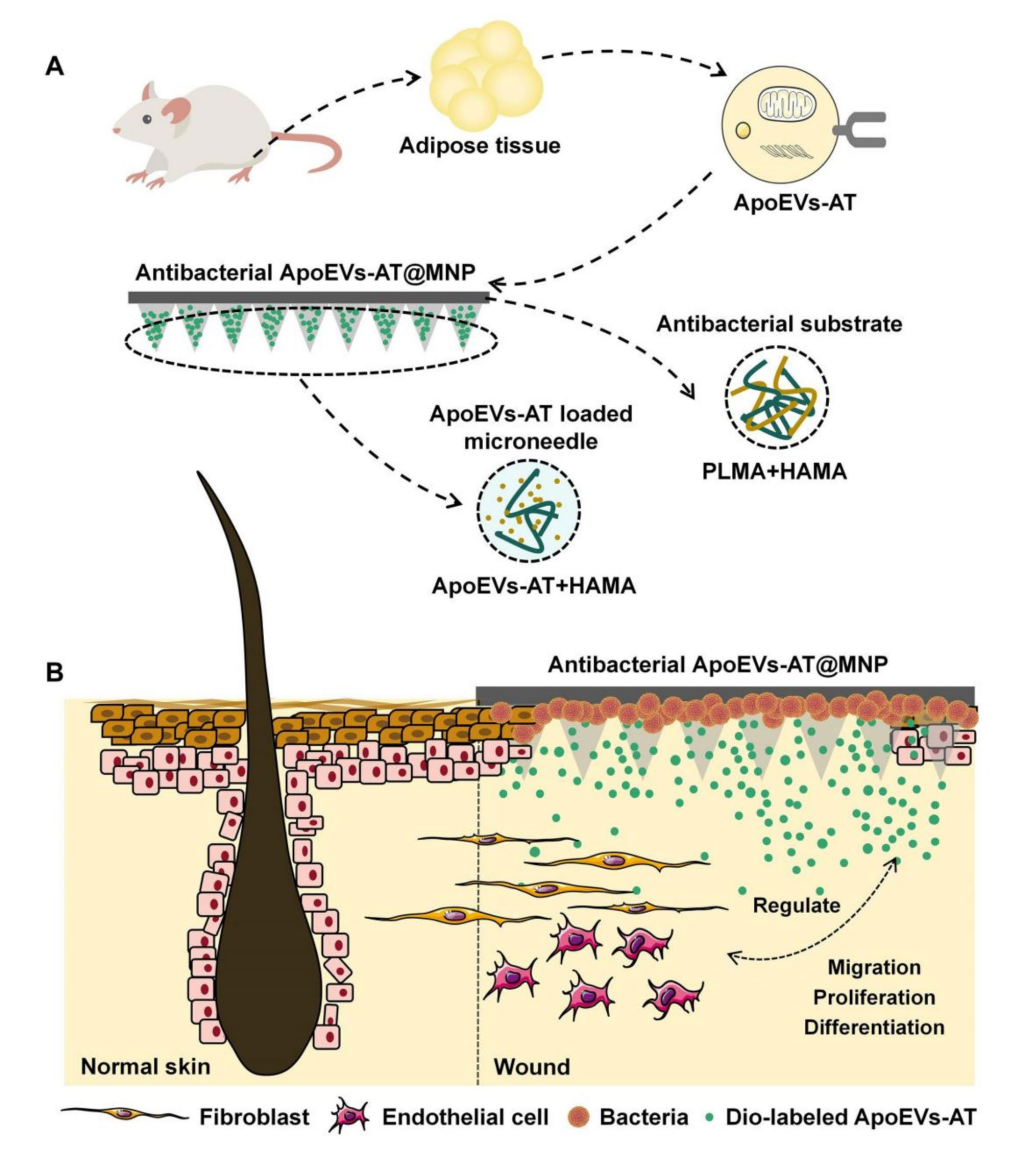
Schematic representation of the design of the experiment. Schematic overview of the **(A)** construction and **(B)** in vivo implantation of ApoEVs-AT@MNP

In this study, a hydrogel microneedle array based on methacrylated hyaluronic acid (HAMA) was developed to control the release of ApoEVs-AT, as shown in Fig. 1A. A hybrid antibacterial hydrogel solution composed of methacrylated polylysine (PLMA) and HAMA was used to fabricate the substrate for the MNP. This resulted in a novel soluble antimicrobial ApoEVs-AT@MNP, with soluble tips that deliver apoptotic vesicles and an antibacterial substrate that inhibits bacterial growth on infected wounds. The MNP system demonstrated appropriate mechanical strength and was able to penetrate the skin, with the tips remaining in the tissue for continuous active release. The entire system is safely biodegradable within the host body. The antimicrobial ApoEVs-AT@MNP not only inhibits bacterial proliferation in infected wounds but also promotes effective, rapid, and high-quality scarless wound healing. ApoEVs-AT@MNP facilitated the rapid formation of mature hair follicles in a regular arrangement within infected wounds just eight days after implantation, a result not observed in previous studies. Therefore, ApoEVs-AT@MNP stands out as an excellent, painless, non-invasive, and highly promising treatment method for infected wounds.

## Materials and methods

### HAMA and PLMA/HAMA preparation

HAMA solutions were created by dissolving different ratios of lyophilized HAMA (50 mg/mL, Engineering for Life, EFL, China) and lithium phenyl-2,4,6-trimethylbenzoylphosphinate (LAP, 25 mg/mL, EFL, China) in α-MEM. PLMA solutions were obtained by solubilizing different ratios of lyophilized PLMA (EFL, China) and LAP (25 mg/mL) in PBS. PLMA solution was subsequently mixed with the HAMA solution to obtain antibacterial hybrid hydrogel with final concentrations of 5% (w/v) HAMA, 5% (w/v) HAMA + 2% (w/v) PLMA, 5% (w/v) HAMA + 4% (w/v) PLMA, 5% (w/v) HAMA + 6% (w/v) PLMA.

### Characterization of HAMA and PLMA/HAMA

#### Assessment of rheological properties

To analyze the rheological behavior of the HAMA and PLMA/HAMA hydrogel, a viscoelasticity analysis was conducted using an HAAKE Viscotester iQ Air (Thermo Scientific). A C35 1°/Ti cone rotator with a truncation gap distance of 1 mm was employed for the experiments. The shear viscosity of the HAMA and PLMA/HAMA hydrogel was determined by performing shear rate sweeps, varying the applied shear rate from 0.1 to 100 s^− 1^ at 25 °C. The dynamic modulus of the hydrogel was determined by frequency sweeps. A value of 0.01 was obtained for the applied strain constant across 0.1–10 Hz at 25 °C.

#### Assessment of antimicrobial properties

To assess the antibacterial characteristics of various ratios of antibacterial hybrid hydrogel, a bacterial suspension with a concentration of approximately 10^6^ CFU/mL was incubated for 24 h with four types of antibacterial hybrid hydrogel after light curing, using a hand-held blue light torch at 405 nm. Following incubation, the bacterial suspension was appropriately diluted, and the inhibition rate was estimated by measurement of the optical density (OD). Additionally, the bacterial suspension, diluted 10^4^ times, was inoculated onto solid nutrient medium surfaces and cultured for 24 h at 37 °C. The Petri dishes were subsequently retrieved, and colony counting was accomplished using the plate counting method to determine antibacterial efficacy. The experiments were conducted in triplicate.

### ApoEVs-AT extraction and labeling

ApoEVs-AT was extracted as reported before [11]. In summary, inguinal adipose tissue was extracted from 4-weel-old Sprague-Dawley rats and sliced into 1–2 cm³ pieces, which were then placed into a suspension culture flask (Wheaton, USA). Apoptosis was induced in the isolated adipose tissue by the addition of serum-free minimum essential medium α (α-MEM, HyClone, USA) with staurosporine (250 nM, STS, Beyotime, China). The mixture was allowed to culture for 2 days at 37 °C in a 5% CO_2_-95% air atmosphere with shaking at 200 rpm. Apoptosis in the adipose tissue was examined using hematoxylin and eosin (H&E) and immunofluorescence (IF) staining with TdT-mediated dUTP nick-end labeling (TUNEL) assays, as previously described [11]. The tissue pieces were then gently removed using sterile gauze, followed by the careful collection of the supernatant. Large extracellular vesicles and tissue debris were removed via centrifugation at 800×g for 10 min at 4 ℃, followed by 2000×g for 15 min at 4 ℃. Further centrifugation of the supernatant at 16,000×g for 30 min at 4 ℃ yielded ApoEVs-AT which were suspended in PBS for subsequent testing. For labeling, 100 µg of ApoEVs-AT suspended in 1 mL of α-MEM were incubated for 30 min with the membrane-labeling dye DiO (1 µg, Life Tech, V22886) at 37 ℃. The labeled ApoEVs-AT were then re-purified by 30 min centrifugation at 16,000×g at 4 ℃.

### ApoEVs-AT identifcation

ZetaView analysis system (Particle Metrix, Germany) was employed to measure the size distribution. To examine the morphological characteristics, ApoEVs-AT were placed on formvar carbon-coated grids, subjected to negative staining with aqueous phosphotungstic acid for 60 s at room temperature, and subsequently imaged using a transmission electron microscope (TEM, Tecnai G2 F20 S-Twin, USA). For detection of phosphatidylserine (PtdSer), 100 µL PBS was used to suspend ApoEVs-AT, 5 µL FITC Annexin V (BD, USA) was added, and the system was allowed to incubate at room temperature for 15 min. A confocal laser scanning microscope (CLSM, Olympus, FV1200, Japan) was used to capture the images. Apoptosis-specific marker proteins were identified using Western blotting. ApoEVs-AT (50 µg) were combined with a 4× loading buffer (Solarbio, China) and heated to boiling for 10 min. SDS-PAGE gel electrophoresis (10% or 15%, 120 V, 90 min) was employed to separate the proteins which were then transferred onto a nitrocellulose membrane. The membrane was subsequently subjected to overnight incubation with primary antibodies, caveolin-1 (1:1000, Sangon Biotech, D161423), Histone H3 (1:1000, Invitrogen, PA5-16,183), cleaved caspase-3 (1:1000, Cell Signaling Technology, #9664) at 4 ℃. Horseradish peroxidase (HRP) conjugated secondary antibodies were subjected to incubation for 2 h at ambient temperature. Highsig ECL Western Blotting Substrate (Tanon, China) was employed for the detection of protein signals by the ImageQuant LAS 4000 mini machine (GE Healthcare, USA). All experiments were conducted at least three times.

### Fabrication of ApoEVs-AT@MNP

The HAMA solution was mixed with ApoEVs-AT to create an active hydrogel solution with a final concentration of 500 µg/mL. Subsequently, 400 µL of the ApoEVs-AT-loaded HAMA hydrogel was transferred to a polydimethylsiloxane (PDMS) mold and placed in a vacuum defoaming machine to remove any foam. The sample was then oven-dried at 35 °C for 5 h. This process was carried out twice to ensure consistency. Next, 300 µL of antibacterial HAMA/PLMA hydrogel solution was added to the PDMS mold. The system was then dried overnight in an oven at 35 °C. Following the drying process, the system was subjected to photocuring using a hand-held blue light torch at 405 nm. The ApoEVs-AT@MNP was then carefully peeled off the mold for further use. The resulting MNP was a square measuring 2 cm on each side and consisted of 20 × 20 microneedle arrays, each with a height of 500 μm and substrate diameters of 200 μm. The base of the microneedle tips was coated with a 5% (w/v) HAMA and 4% (w/v) PLMA mixture, forming the substrate part of the ApoEVs-AT@MNP. Each ApoEVs-AT@MNP contains 400 µg of ApoEVs-AT, the effective dose identified in previous studies.

### Characterization of MNP

#### Morphology observation

The MNP’s bright-field images and overall morphology were examined using a stereomicroscope (Olympus, Japan), and its surface morphological features were observed using SEM (Phenom, Netherlands). Fluorescent MNP, prepared by mixing HAMA with green fluorescent dyes, were visualized using CLSM (Olympus, FV1200, Japan).

#### Mechanical property

The MNP was evaluated for its mechanical strength using a displacement-force test station (Hengyi, China) equipped with a 50 kg load sensor. Microneedle tips were positioned vertically on a rigid stainless-steel platform, and the sensor was lowered at a rate of 0.1 mm/s. Initially, the sensor and the microneedle tips were 1 cm apart. Measurement of displacement and force commenced when the sensor made contact with the microneedle tips, with the speed adjusted to 0.01 mm/s, and continued until the sensor had traveled 800 μm.

#### In vitro ApoEVs-AT release profile test

Each ApoEVs-AT@MNP sample was immersed in 5 mL of PBS (pH 7.4) at 37 °C. At specified time points (1, 2, 3, 4, 5, 6, 7 and 8 days), 20 µL of the release medium was collected. The experiment was performed in a water bath at 37 °C. Using a BCA protein assay kit (Thermo Scientific), the total protein concentration released from the ApoEVs-AT@MNP was measured according to the manufacturer’s protocol. Absorbance was recorded with a microplate spectrophotometer (Tecan, Switzerland) at 562 nm. The experiments were repeated at least thrice.

### Biological effects of ApoEVs-AT@MNP

The conditioned medium was obtained by incubating ApoEVs-AT@MNP in PBS for 72 h.

#### Cellular uptake

For cellular uptake studies, 5 × 10^4^ fibroblasts or ECs were initially plated in a confocal culture dish (NEST, China). The cells were allowed to incubate with a conditioned medium for 24 h. Following incubation, cells were fixed at room temperature with neutral paraformaldehyde (4%, Biosharp, USA) for 10 min and permeabilized with Triton X-100 (0.05%, Sigma-Aldrich, USA) for 15 min at ambient temperature. Alexa Fluor 555 Phalloidin (1:200, Invitrogen, A34055-300U) was used to stain the cytoskeleton for 20 min at room temperature, and DAPI (1:1000, Solarbio, C0050) was used to stain the nuclei for 5 min at room temperature. Images were acquired using CLSM (Olympus, FV1200, Japan). All procedures were carried out in triplicate.

#### CCK-8 assay

Fibroblasts proliferation was assessed using the Cell Counting Kit-8 (CCK-8, Dojindo, Japan). Fibroblasts were plated at a density of 1 × 10^3^ cells per well in 96-well plates and left to incubate at 37 °C overnight. The cells were subsequently cultured with a conditioned medium and α-MEM as a blank control. At days 1 through 7, 10 µL of CCK-8 solution was added to individual wells and incubated at 37 °C for 90 min. Absorbance values were recorded using a Multiskan Go Spectrophotometer (Thermo Fisher Scientific, Waltham, MA, USA). Similarly, the proliferation of ECs was measured using the same protocol, with observations taken on days 1 through 5. All experiments were performed in triplicate.

#### Transwell assay

Fibroblasts migration was assessed using Transwell assays. The upper compartment of a Chemotaxicell Chamber (8 μm, Osaka, Japan) was inoculated with 1 × 10^4^ cells while the conditioned medium was added to the lower compartment. α-MEM without ApoEVs-AT served as the control. Following the passage of 24 h, the chamber was washed with PBS. Using a cotton swab, the non-migrated cells on the upper side of the membrane were removed, while 0.5% crystal violet was employed to fix and stain the remaining cells for 10 min. Cells were counted in the central, top, bottom, left, and right fields of view per filter and averaged to determine the number of migrated cells. The same procedure was used to measure ECs migration. All experiments were performed in triplicate.

#### Tube formation assay

ECs were pretreated with a conditioned medium and α-MEM without ApoEVs-AT for 2 days. The cells were then seeded at 10^4^ cells per well into Matrigel-coated 96-well plates. After 6 h, an inverted microscope (Olympus, Japan) was employed to capture phase-contrast images. Image Pro Plus software was employed to determine the number of nodes and the length of the tubular structures in each field. All experiments were repeated at least thrice.

#### Adipogenic differentiation assay

To examine fibroblasts differentiation induced by ApoEVs-AT released from ApoEVs-AT@MNP, a 24-well plate was seeded with fibroblasts at 1 × 10^5^ cells per well. The fibroblasts were categorized into two groups: a blank group and an ApoEVs-AT@MNP group. To ensure consistent treatment, the culture medium was replaced every 2 days. Following 20 days of incubation, cells were collected. Total RNA was extracted using RNAiso Plus (TaKaRa Biotechnology, Japan) and reverse transcribed into cDNA with the RevertAid First Strand cDNA Synthesis Kit (Thermo Scientific, USA). The cDNA was then amplified using SYBR Premix ExTaq (TaKaRa Biotechnology, Japan) on a QuantStudio 6 Flex Real-Time PCR System (Life Technologies, China). The PCR conditions were 95 °C for 2 min, followed by 44 cycles of 95 °C for 5 s and 60 °C for 30 s (*n* = 3). The expression of PPARγ2, C/EBPα, Adiponectin, and FABP4 was examined to evaluate adipogenesis in fibroblasts. Primer sequences are provided in Supplementary Table S1. All experiments were performed in triplicate.

### Infected wound healing model

An intraperitoneal injection of 1% pentobarbital sodium (10 mL/kg) was used to induce general anesthesia on 4-week-old male Sprague-Dawley rats (*n* = 4) before conducting any surgical procedures. After shaving and disinfecting the dorsal area with 75% ethanol, a pair of circular full-thickness skin wounds, each 2 cm in diameter, were created by resecting along markings drawn with a pen. A concentrated suspension of *Staphylococcus aureus* was applied to the wound and recorded as day 2. The wound was examined after 48 h, recorded as day 0. If the wound exhibited suppuration, the model was considered successful. The wounds were assigned to two groups: ApoEVs-AT@MNP group (left); and blank group (right). ApoEVs-AT@MNPs were inserted in the relevant group on days 0 and 8, and covered with a transparent dressing (Fig. 1B). The blank group was subcutaneously injected with 100 µL of PBS around the wounds on days 0 and 8. The wounds were photographed digitally on days 0, 4, 8, 12, and 16. ImageJ 1.53a software was used to measure the wound areas. On days 8 and 16, the rats were euthanized (*n* = 3 per time point) through an overdose of anesthesia, and the tissue specimens were collected for additional analysis.

### Histology and IF staining

For histological evaluation, wound tissues collected on days 8 and 16 were subjected to fixation with paraformaldehyde (4%, Biosharp, USA) overnight. A gradient of ethanol was then used to dehydrate the samples, which were cleared in xylene, followed by their embedding in paraffin and cutting into sections of 6 μm thickness. The obtained sections were then subjected to staining with hematoxylin and eosin (H&E) (Solarbio, China) and Masson’s trichrome stain (Baso, China). The stained sections were examined using optical microscopy (Olympus, Japan).

For a further assessment of the specific structure of the specimens, 5% bovine serum albumin (BSA, Sigma-Aldrich, USA) was used to block the sections near the center of the wound at room temperature for 2 h. They were then allowed to incubate overnight with primary antibodies at 4 °C. Primary antibodies, Perilipin A (1:200, Abcam, ab3526) and CD31 (1:200, Abcam, ab24590), were respectively employed to mark adipocytes and blood vessels. Primary antibodies, collagen 1 (Col 1, 1:200, Abcam, ab270993), alpha-smooth muscle actin (α-SMA, 1:200, Abcam, ab5694), and collagen 3 (Col 3, 1:200, Abcam, ab184993), were utilized to mark various kinds of fibers. Subsequently, the secondary antibodies, goat anti-rabbit 555 (1:200, Invitrogen, A21428) and goat anti-mouse 488 (1:200, Invitrogen, A11008), were subjected to incubation for 1 h at 37 ℃. DAPI (1:1000, Solarbio, C0050) was then employed to mark the nuclei at room temperature for 5 min. Images were captured by CLSM (Olympus, FV1200, Japan) and ImageJ 1.53a software was employed for their analysis (*n* = 3).

### Statistical analysis

Each experiment was repeated independently at least three times to ensure the reproducibility of the data. All numerical data are presented as means ± SD. Statistical significance was analyzed using GraphPad Prism 9.0.0 software. Paired and unpaired t-tests and one-way and two-way ANOVA were used to assess significant differences. A value of *p* < 0.05 was considered statistically significant. Statistical significance: *p < 0.05, **p < 0.01, ***p < 0.001. ****p < 0.0001.

## Results

### Preparation and properties of antibacterial hybrid hydrogel

Hyaluronic acid (HA), a natural glycosaminoglycan polymer, is a principal constituent of the extracellular matrix in animal tissues. It plays a vital role in various biological processes, including cell proliferation, differentiation, inflammation, morphogenesis, and wound healing [20]. HAMA was obtained by the introduction of methacrylate groups onto the molecular chain of HA, endowing it with photocuring capability [21]. HAMA hydrogel exhibit high water absorption capacities and good biocompatibility. Epsilon-polylysine (ε-PL), on the other hand, is an antibacterial peptide produced by Streptomyces [22]. It possesses broad-spectrum antibacterial activity, good biocompatibility, and biodegradability, thus effectively circumventing the limitations of traditional antibacterial drugs or metal ions (such as Ag^+^) [22, 23]. PLMA, a polylysine modified with double bonds, can be cross-linked and solidified into a gel using a photoinitiator and exposure to UV or visible light.

**Fig. 2:**
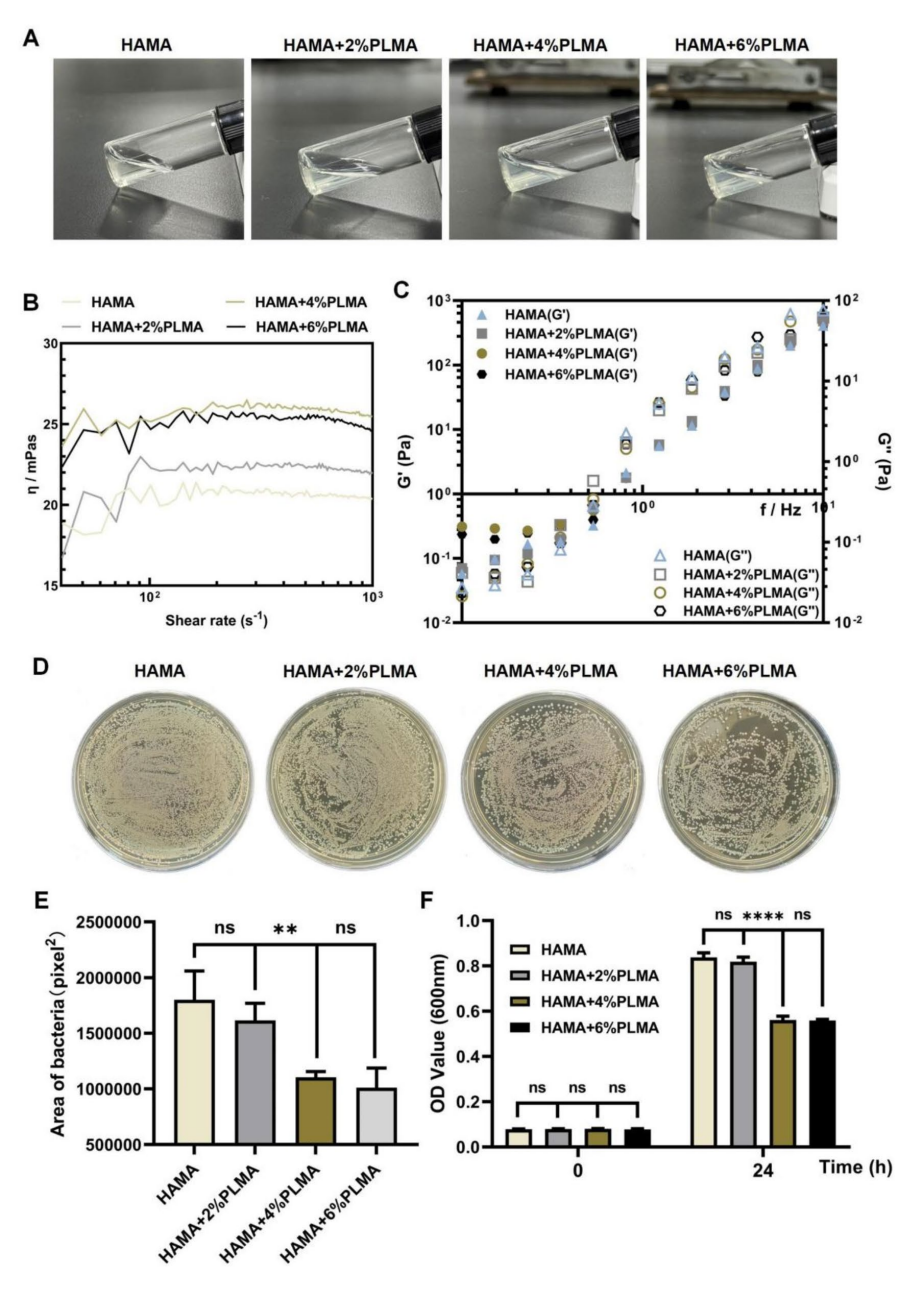
Fabrication and characterization of HAMA and HAMA/PLMA. **(A)** Macroscopic appearance of HAMA/PLMA antibacterial hybrid hydrogel with different PLMA concentrations. **(B)** Viscosity and (**C**) storage G′ and loss moduli (G′′) determined at 25 ℃. **(D-F)** The antibacterial properties of HAMA and PLMA/HAMA. In vitro antibacterial activity of MN. **(D)*** S. aureus* proliferation following co-culture with HAMA and PLMA/HAMA. Corresponding quantitative analysis **(E)** and OD value counting **(F)** of bacteria counting on agar plates in (**D**)

Therefore, a mixed hydrogel solution was initially prepared with varying concentration ratios of PLMA and HAMA. After photocrosslinking, the hybrid hydrogel did not exhibit any significant change in terms of its transparency, with the increase in PLMA concentration (Fig. 2A). Rheology serves as an effective method for assessing the mechanical properties of the hydrogel. Our findings indicated that both the viscosity and dynamic modulus of the hydrogel demonstrated an increase with the addition of PLMA. At a PLMA concentration of 4%, the mixed hydrogel solution exhibited the highest viscosity, enhancing its suitability for conforming to the micro-needle substrate and the wound surface (Fig. 2B). The elastic and viscous behavior of the hydrogel is respectively associated with the storage and loss moduli [24]. Across the entire frequency range, the storage moduli (G′) exceeded the loss moduli (G′′) in all four hybrid hydrogel (Fig. 2C). This observation suggested that the HAMA/PLMA hybrid hydrogel could withstand deformation and demonstrated the elastic properties characteristic of a strong hydrogel network [24]. The solution employed for the preparation of the microneedle array should have a relatively low viscosity to adequately fill the PDMS mold, facilitating the easy removal of the hydrogel microneedles from the mold after curing [25]. The addition of PLMA significantly increases the viscosity of the HAMA hydrogel solution. To maintain the integrity of the microneedle array, hydrogel microneedles based on HAMA were employed as a carrier for the controlled release of ApoEVs-AT. Furthermore, the MNP substrate was fabricated using a hybrid antibacterial hydrogel solution composed of both PLMA and HAMA.

To further evaluate the antibacterial properties of HAMA/PLMA hybrid hydrogel, its antibacterial potential was investigated against *S. aureus*, which is a common pathogen causing wound infections [26, 27]. As shown in Fig. 2D and E, no significant antibacterial effect was observed at a PLMA concentration of 2%. However, at PLMA concentrations of 4% and 6%, the growth and proliferation of high-concentration *S. aureus* were effectively inhibited, with no statistical difference between these two groups. Additionally, bacterial suspensions co-incubated for 0 and 24 h were tested for their OD values (Fig. 2F), which were consistent with the colony counting results. According to previous reports, high concentrations of PLMA can inhibit cell adhesion, growth, and proliferation. To ensure the cell compatibility of the hybrid antibacterial hydrogel solution, a PLMA concentration of 4% was selected as the substrate for the microneedle patch.

### Extraction and identification of ApoEVs-AT

**Fig. 3:**
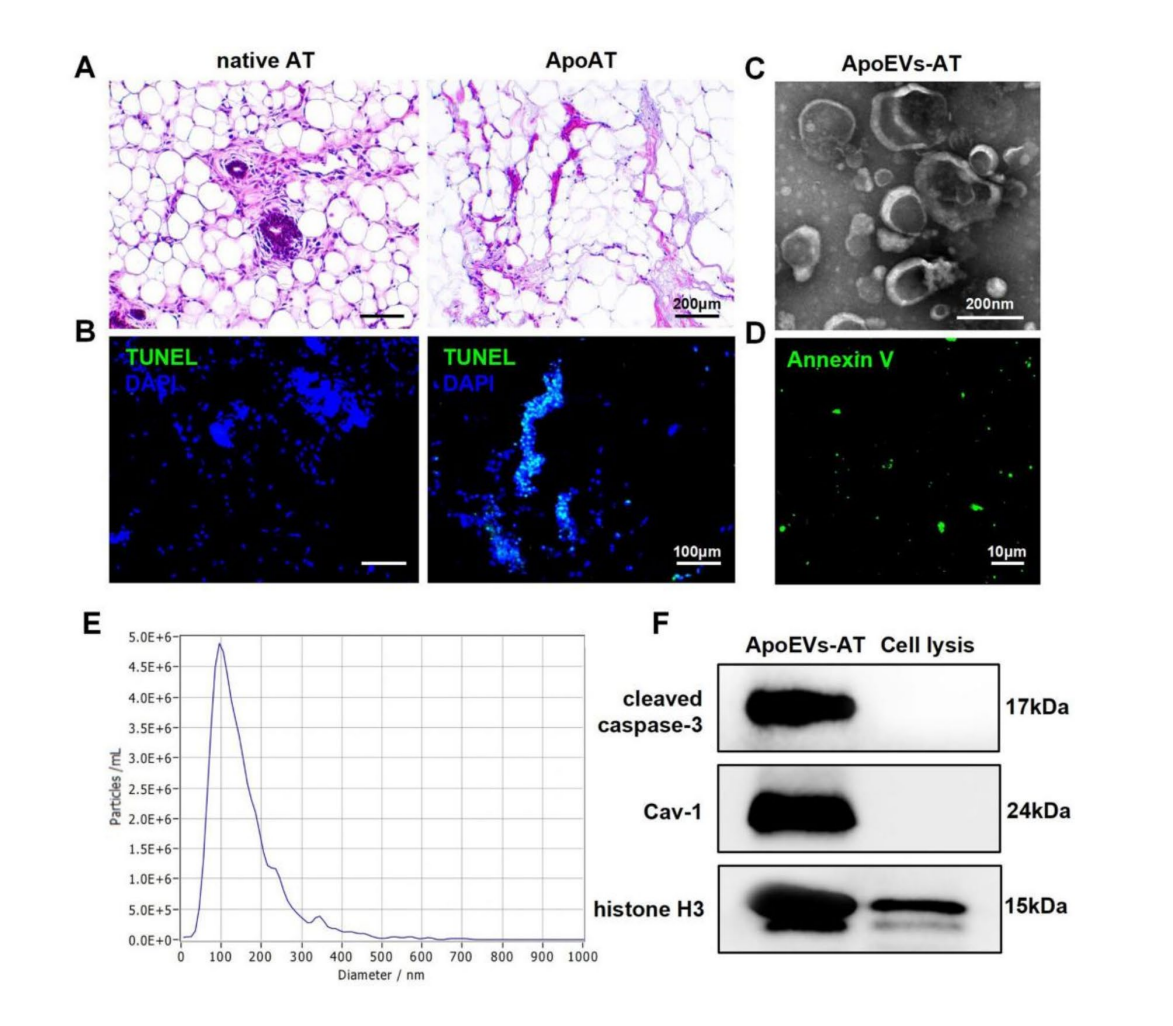
Preparation and characterization of ApoEVs-AT. Representative images of native adipose and apoptotic adipose tissues with **(A)** H&E staining. Scale bar, 200 μm. **(B)** TUNEL staining of native adipose tissue and apoptotic adipose tissue. Blue: DAPI-stained nuclei; Green: TUNEL-stained apoptotic cells. Scale bar, 100 μm. **(C)** TEM micrographs of ApoEVs-AT. Scale bar, 200 nm. **(D)** Annexin V staining of ApoEVs-AT. (Green: Annexin V-stained ApoEVs-AT. Scale bar, 10 μm. **(E)** Size distribution of ApoEVs-AT. **(F)** Western blotting of marker proteins, cleaved caspase-3, caveolin-1 (Cav-1), histone H3

Apoptotic vesicles were extracted from adipose tissue using echelon centrifugation. H&E and TUNEL staining was used to evaluate the structure and apoptosis of the adipose tissue. The apoptotic adipose tissue displayed an irregular and loose structure in comparison to native adipose tissue, (Fig. 3A). Significant apoptosis was observed in the apoptotic adipose tissue through TUNEL staining, whereas no TUNEL-stained nuclei were seen in the native adipose tissue (Fig. 3B). For further characterization of the ApoEVs-AT, their size distribution, morphology, and specific markers were analyzed. TEM analysis demonstrated that ApoEVs-AT possessed a double-membrane spherical structure (Fig. 3C). Immunofluorescence imaging showed that ApoEVs-AT were positive for the apoptosis-specific marker phosphatidylserine (PtdSer), as indicated by Annexin V staining (Fig. 3D). Nanoparticle tracking analysis (NTA) demonstrated that most ApoEVs-AT ranged from 25 to 500 nm in size, with a median particle size of 122.1 nm (Fig. 3E). Western blot analysis confirmed the presence of cleaved caspase-3, caveolin-1 (Cav-1), and histone H3 in ApoEVs-AT, which are apoptosis-specific marker proteins (Fig. 3F) [9]. The HAMA solution was mixed with ApoEVs-AT to achieve an active hydrogel solution.

### Preparation and characterization of ApoEVs-AT@MNP

**Fig. 4:**
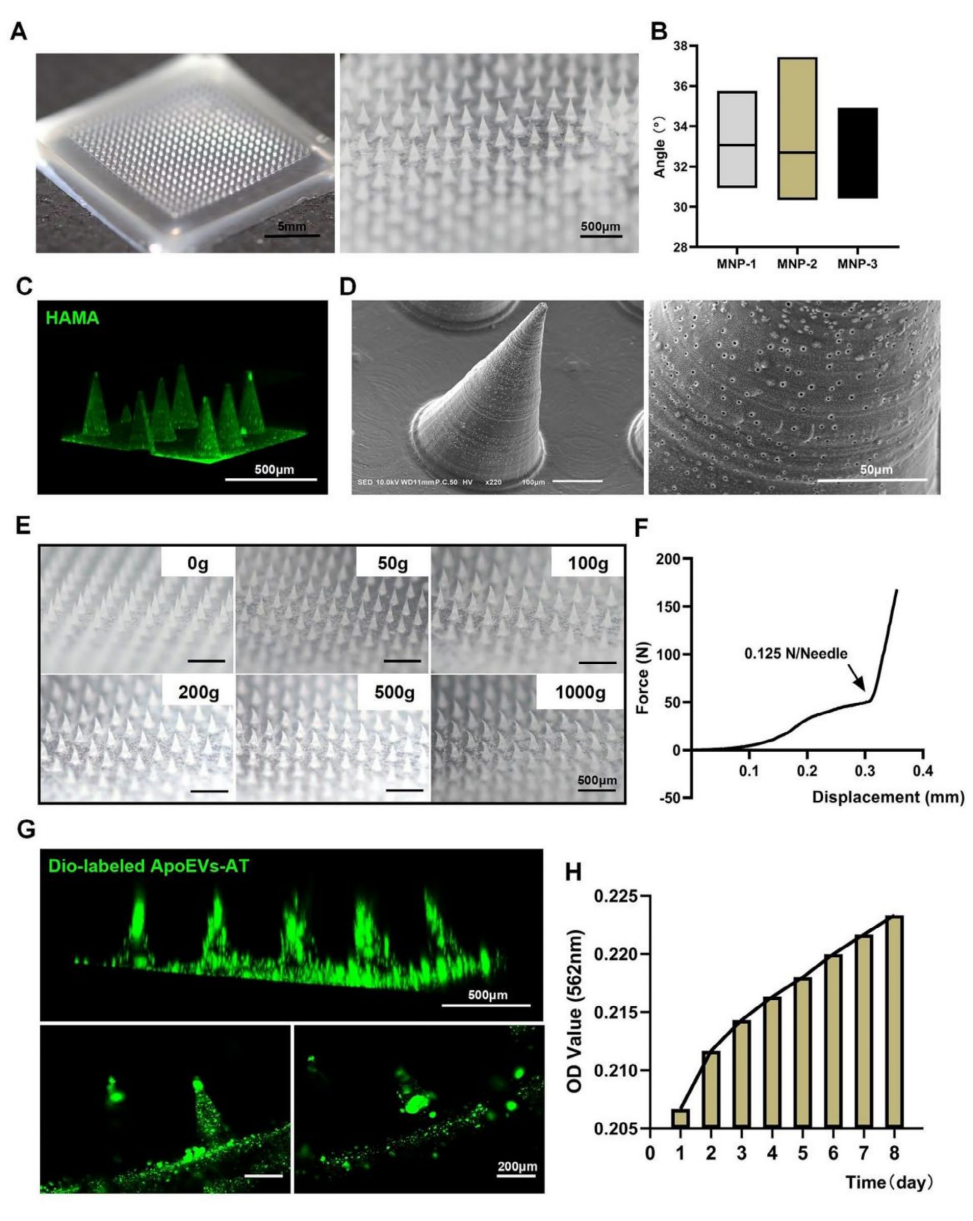
Identification of material properties and drug loading capacity of ApoEVs-AT@MNP. **(A)** Physical picture of HAMA@MNP. Scale bar, left, 5 mm. Scale bar, right, 500 μm. **(B)** Tip angle statistics of HAMA@MNP. **(C)** Green fluorescent-labeled HAMA@MNP. Scale bar, 500 μm. **(D)** SEM images of HAMA@MNP. Scale bar, left, 100 μm. Scale bar, right, 50 μm. **(E)** The deformation of microneedle tips under varying loads. Scale bar, 500 μm. **(F)** The stress-displacement curves of tip deformation in axial compression experiments. **(G)** Dio-labeled ApoEVs-AT@MNP was visualized using a confocal microscope. Scale bar, top, 500 μm. Scale bar, below, 200 μm. **(H)** Protein release assays of ApoEVs-AT@MNP via BCA test

To evaluate the potential of HAMA hydrogel-based soluble MNP as efficient vesicle delivery vehicles, drug-free HAMA@MNP was fabricated and subjected to comprehensive material property evaluations. The resulting HAMA@MNP consist of 20 × 20 microneedle arrays with a height of 500 μm and substrate diameters of 200 μm. Gross digital photographs in Fig. 4A showed the intact structure of both the HAMA@MNP and the microneedle tips. Statistical analysis of the tip angle within the microneedle matrix indicated a range of 30° to 38° (Fig. 4B), facilitating easy insertion into the skin. This sharp pyramidal structure ensures that the microneedles can be quickly and accurately inserted into the deep skin layers noninvasively [18, 28, 29]. To provide a more intuitive representation of the HAMA@MNP microstructure, fluorescent-labeled HAMA was used to construct the HAMA@MNP. Three-dimensional visualization by CLSM confirmed the integrity of the tips (Fig. 4C). SEM images revealed the presence of pores on the surface of the HAMA@MNP, facilitating the release of ApoEVs-AT (Fig. 4D). When the microneedle array pierces the skin, it rapidly absorbs interstitial fluid into the grid, allowing for gradual expansion of the material and enlargement of the micropores on the surface for drug delivery. The physical properties of HAMA@MNP were evaluated through axial compression experiments. Results showed that within a resistance weight range of 0-10^3^ g, the HAMA@MN maintained a relatively stable morphology (Fig. 4E). As illustrated in Fig. 4F, individual needle tips exhibited a maximum mechanical strength of approximately 0.125 N per needle, exceeding the required insertion force to penetrate the skin barrier. The general overview of the HAMA@MN axial compression experiment was observed using a universal machine (Fig. S1). Earlier studies have shown that the minimum amount of force required to efficiently penetrate the skin is 0.03 N [30]. The aforementioned results preliminarily suggest that the HAMA-based microneedle array possesses favorable material characteristics, thus exhibiting promising potential as a carrier for ApoEVs-AT.

A hydrogel solution was subsequently prepared by combining Dio-labeled ApoEVs-AT with a HAMA hydrogel solution. After the ApoEVs-AT-loaded HAMA@MNP was fabricated, it was placed under CLSM scanning, showing a strong drug-loading capacity of the HAMA@MNP (Fig. 4G). CA detection demonstrated that the ApoEVs-AT could be gradually released from The top image shows the projection in the XZ axis after 3D reconstruction, while the bottom images are tomographic images, indicating that the Dio-labeled vesicles were mostly concentrated in the tip and middle part of the needle body with minimal fluorescence in the MNP substrate. It is important that during MNP fabrication, the hydrogel should not be dried completely with each heating and concentration process after sample addition, as this could result in incomplete microneedle arrays and separation of the array fom the substrate. After the second addition of ApoEVs-AT to the HAMA hydrogel and subsequent heating and concentrating, we deliberately left a few ApoEVs-AT-loaded HAMA hydrogels at the bottom of the PDMS mold before the addition of the hybrid PLMA/HAMA hydrogel solution as the substrate to guarantee the integrity of the structure. Bthe HAMA@MNP over 8 days (Fig. 4H). The drug loading of ApoEVs-AT in the ApoEVs-AT@MNP was then calculated and adjusted based on the slow-release rate of the HAMA@MNP, following a previous study on effective treatment doses for skin wounds using ApoEVs-AT. Each resulting ApoEVs-AT@MNP contained 400 µg of ApoEVs-AT, thereby facilitating subsequent in vitro tests and in vivo transplantation.

### Regulation of fibroblasts and ECs behavior by ApoEVs-AT@MNP

**Fig. 5:**
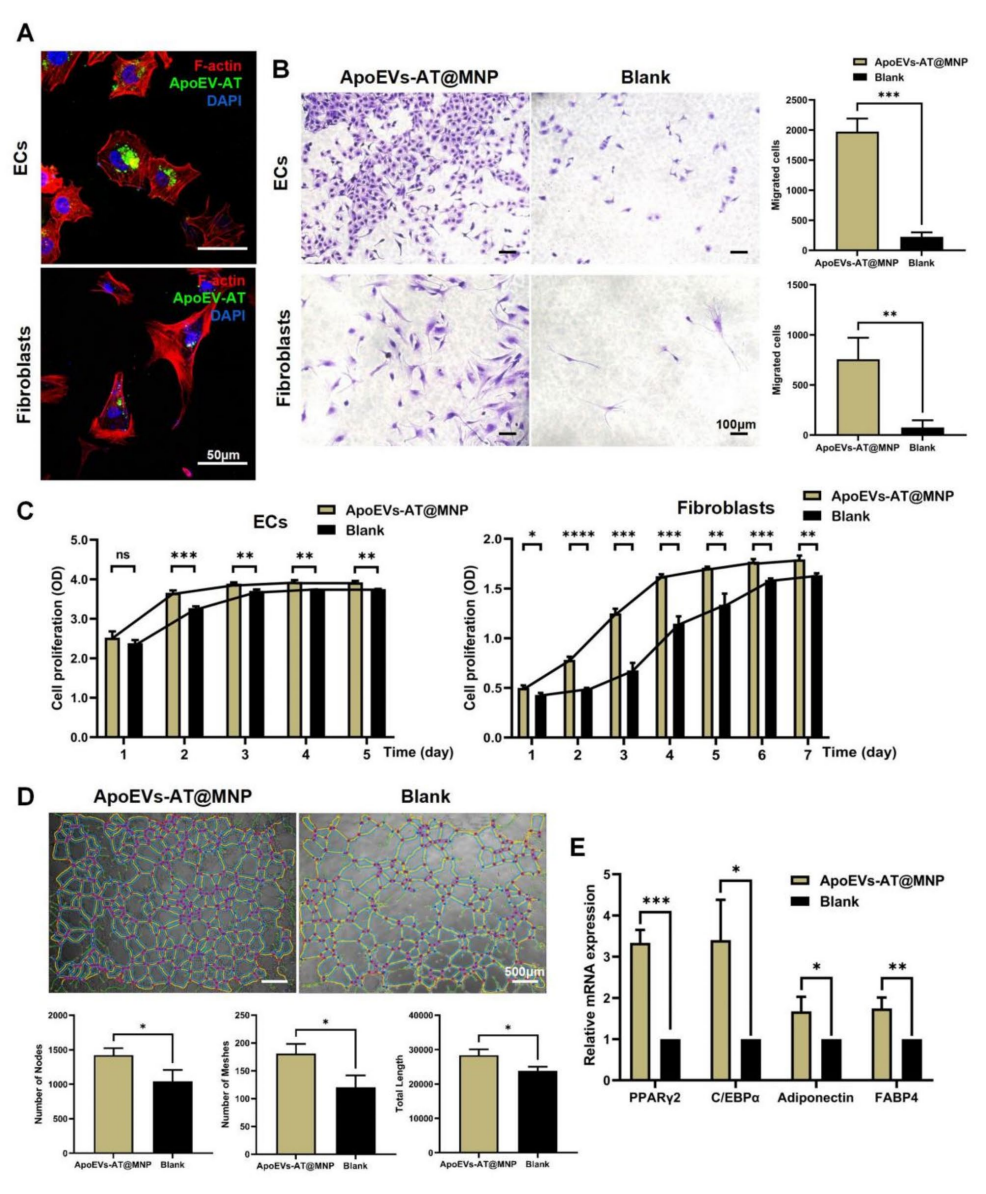
ECs and fibroblasts behavior regulated by ApoEVs-AT released by ApoEVs-AT@MNP. **(A)** Analysis of the uptake of ApoEVs-AT by ECs and fibroblasts. Blue: DAPI-stained nuclei, Green: Dio-labeled ApoEVs-AT, Red: phalloidin-stained cells. Scale bar, 50 μm. **(B)** Representative images of cells migrating in transwell assays, along with quantification of the number of migrated cells per field of view. Scale bar, 100 μm. **(C)** ECs and fibroblasts proliferation measured by CCK-8 assays. **(D)** Representative images of the tube-like structure formed by ECs. Scale bar, 500 μm. Number of nodes, meshes, and total length per field of view were analyzed below. **(E)** qRT-PCR based detection of the relative expression of adipogenic-related genes (FABP4, Adiponectin, C/EBPα, and PPARγ)

To investigate the impact of ApoEVs-AT@MNP on key cells involved in skin regeneration, fibroblasts and ECs were selected and co-cultured with Dio-labeled ApoEVs-AT@MNP in vitro. After 24 h, both fibroblasts and ECs successfully internalized the Dio-labeled ApoEVs-AT (Fig. 5A), indicating their ability to incorporate ApoEVs released by the ApoEVs-AT@MNP. The conditioned medium was then collected by incubating ApoEVs-AT@MNP in PBS for 48 h and was applied to both cell types. The CCK-8 assay demonstrated that the conditioned medium significantly enhanced the proliferation of fibroblasts and ECs compared to the blank control group (Fig. 5B). Micrographs from the Transwell assay along with statistical analysis revealed that after 12 h, the conditioned medium effectively promoted the migration of both fibroblasts and ECs (Fig. 5C). Furthermore, the experimental results demonstrate that the introduction of endothelial cell-conditioned medium into a tube significantly promotes angiogenesis, as evidenced by increases in the number of nodes, grid formations, and total length (Fig. 5D). Additionally, following the treatment of fibroblasts with the conditioned medium for 20 days, the expression of adipogenesis-related genes—PPARγ, C/EBPα, FABP4 and Adiponectin—was assessed using qRT-PCR. The results showed that the expression of these adipogenesis-related genes was markedly enhanced by ApoEVs-AT (Fig. 5E). Overall, by releasing ApoEVs-AT, ApoEVs-AT@MNP effectively modulates the cellular behaviors of fibroblasts and endothelial cells, providing a cytological basis for potential wound treatment in experimental models.

### Antimicrobial ApoEVs-AT@MNP accelerated healing of full-thickness infected skin wounds

**Fig. 6:**
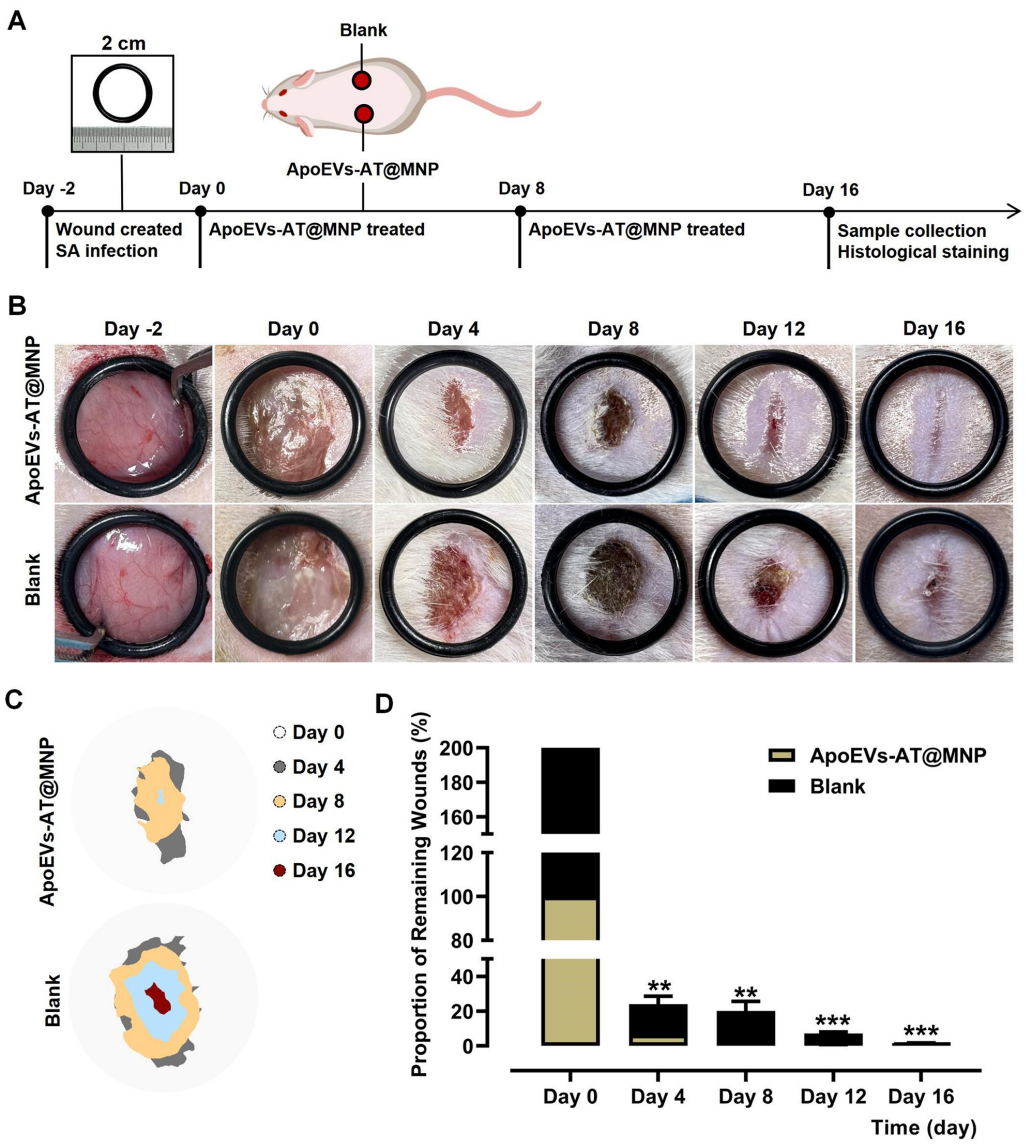
Accelerated healing of infected wounds by ApoEVs-AT@MNP. **(A)** Diagram illustrating the procedural workflow. **(B)** Representative photographs showing wound sites on days − 2, 0, 4, 8, 12, and 16. Diameter of the circle = 2 cm. **(C)** Graphical representation of wound areas at various time points, with initial wounds marked by dotted circles and colored regions indicating wound areas at subsequent time points. **(D)** Analysis of unhealed wound areas, expressed as a percentage of the initial wound size. *n* = 3. Calculation: percentage of unhealed area at day X / initial wound area at day 0 × 100%

As depicted in Fig. 6A, the efficacy of antimicrobial ApoEVs-AT@MNP in promoting the healing of full-thickness skin wounds infected with *Staphylococcus aureus* was assessed. Two circular wounds, each 2 cm in diameter, were created on the dorsal side of individual rats and inoculated with a concentrated suspension of *Staphylococcus aureus* on day − 2. The formation of pus at the wound site, observed 48 h later, confirmed the successful establishment of the infected wound model. This day was designated as day 0. Antimicrobial ApoEVs-AT@MNP were applied once to the left infected wound on day 0 and again on day 8, while the right wound served as a blank control. The wounds were digitally photographed on days 0, 4, 8, 12, and 16 to document the rate of healing, as shown in Fig. 6B. Pattern graphs were generated to delineate the wound edges, with the dotted circle indicating the initial wound boundary on day 0 and the unhealed areas at various time points were depicted in different colors (Fig. 6C). The wound area percentage was calculated using the formula: unhealed wound area at day X / initial wound area at day 0 × 100%. The analysis results indicated that a significant level of antibacterial efficacy and rapid wound healing was achieved with antimicrobial ApoEVs-AT@MNP, with only 6.47% of the wound area remaining unhealed by day 4 (Fig. 6D). A wound healing rate of 99.55% was reached by day 12, while complete healing was not achieved by day 16 in the blank control group. Additionally, some wounds in the blank group showed aggravated infection on day 4, even after routine disinfection (Fig. S2).

### Antimicrobial ApoEVs-AT@MNP improved the quality of nascent tissues

**Fig. 7:**
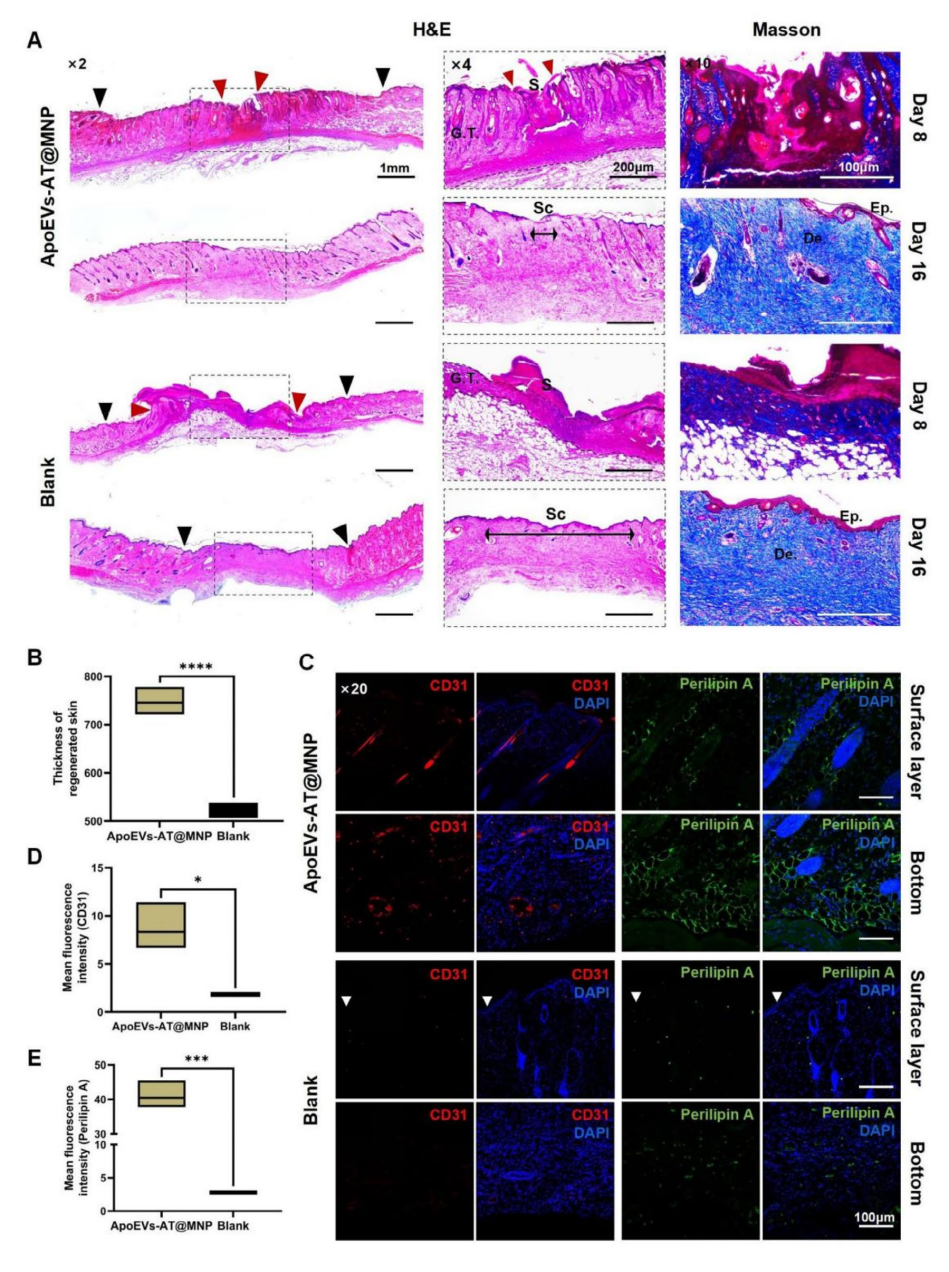
Histological effects of ApoEVs-AT@MNP on infected wound healing. **(A)** H&E and Masson staining of infected wounds in various treatment groups on days 8 and 16. G.T.: granulation tissue; S.: scab; Sr: scar; Red arrow: boundary of the remaining wound; Black arrow: the boundary of the granulation tissue. Scale bar, left, 1 mm. Scale bar, middle, 200 μm. Scale bar, right, 100 μm. **(B)** Quantitative assessment of tissue width across various treatment groups on day 16. **(C)** The expression of CD31 and Perilipin A in the surface layer and at bottom of neogenesis tissues. **(D**, **E)** Quantitative assessment of CD31 **(D)** and Perilipin A **(E)** expression in newly formed tissues across various groups on day 16. Scale bar, 100 μm

**Fig. 8:**
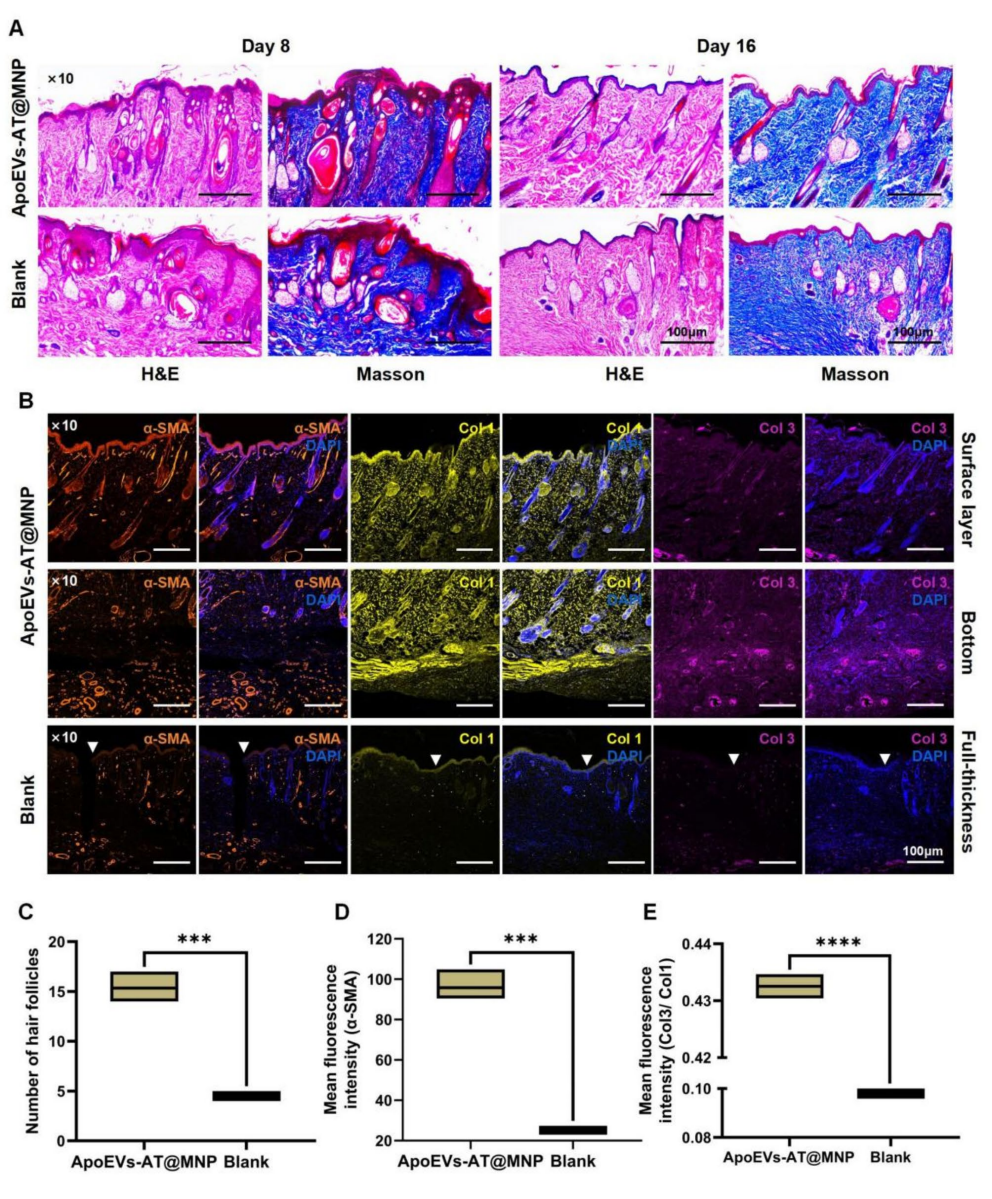
Histological effects of ApoEVs-AT@MNP of regenerated dermal appendages. **(A)** H&E and Masson staining of the regenerated dermal appendages in different groups on days 8 and 16. Scale bar, 100 μm. **(B)** Col 1, α-SMA, and Col 3 expression in the surface layer and bottom of neogenesis tissues in the ApoEVs-AT-MN group and full-thickness of neogenesis tissues in the blank group. Scale bar, 100 μm. **(C)** Quantitative analysis of follicle counts in various groups on day 16. **(D**,** E)** Quantitative assessment of α-SMA **(D)** and Col 3/Col 1 **(E)** expression in newly formed tissues across various groups on day 16

Besides the healing rate, the quality of skin regeneration was a key area of investigation. Although the regeneration of skin structure was optimal, the majority of wounds ultimately formed scars. To assess wound healing quality, the granulation tissue in unhealed wounds was first examined, as this significantly influences the quality of the healed skin. Skin samples collected on day 8 were sectioned and stained for analysis. As illustrated in Fig. 7A, H&E, and Masson’s trichrome staining showed that the antimicrobial ApoEVs-AT@MNP accelerated re-epithelialization. In particular, the zone without epithelization was smaller in the experimental group compared to the control group, consistent with observations from digital photographs (Fig. 8B). The dermis of the nascent tissues in the experimental group displayed a well-organized arrangement of new skin appendages, including hair follicles, sebaceous glands and sweat glands. The central unhealed region of the wound was covered by granulation tissue with only a small scab, as shown in Fig. 7A. Furthermore, the ApoEVs-AT@MNP-treated wounds were fully healed in 16 days, leaving only a small, slender scar. The regenerated skin featured numerous new hair follicles, hairs, and sebaceous glands, closely resembling natural skin, and exhibited extensive infiltration of large-diameter blood vessels, as depicted in Fig. 7A.

In the blank group, the majority of the wound area was covered by granulation tissue and a substantial scab shell, as shown in Fig. 7A. Only a few irregular skin-like appendages were observed at the wound’s periphery. Moreover, complete healing was not achieved within 16 days. Scabs remained, and the scar area was predominantly occupied by fibrous tissue, with only minimal irregular skin attachments at the wound edges, as illustrated in Fig. 7A. No significant invasion of skin vasculature was observed, though a small amount of newly formed microvascular growth was detected at the base of the dermis of the skin.

The statistical analysis of the thickness of newly formed skin in both groups was conducted. In the ApoEVs-AT@MNP group, the average thickness of regenerated tissue was found to be 745.61 μm, whereas that in the blank group was recorded as 529.38 μm. This difference was found to be statistically significant (Fig. 7B). The findings are consistent with those depicted in the digital photograph.

Furthermore, we conducted IF staining and semi-quantitative analysis on tissue sections of day 16, revealing that the ApoEVs-AT@MNP group exhibited a substantial expression of CD31, the marker protein for angiogenesis, and in contrast, the blank group showed negligible expression (Fig. 7C and D), which is consistent with the aforementioned histological staining results. Additionally, previous studies have highlighted the crucial role of adipocytes in skin regeneration and development [21, 22]. Adipocytes were interestingly observed in both the surface and bottom layers of new skin in the ApoEVs-AT@MNP group, as indicated by perilipin A staining. In contrast, the blank group showed nearly no adipocytes in the surface layer and only a small amount of protein in the bottom layer (Fig. 7C). These findings were further supported by fluorescence quantitative statistics (Fig. 7E). Thus, the results unequivocally confirm that antimicrobial ApoEVs-AT@MNP effectively enhance granulation tissue quality during the healing process of infected skin wounds.

### ApoEVs-AT@MNP regulated the rearrangement and maturation of the new skin appendages

For the restoration of normal structure, function, and mechanical strength in injured skin, the regeneration of skin appendages, such as sebaceous glands and hair follicles is necessary [31]. To further assess the quality of new skin and the regeneration of skin appendages, the area near the scar boundary was examined. HE and Masson trichrome staining demonstrated that ApoEVs-AT@MNP demonstrated the ability to rapidly regenerate skin appendages and enabled their organized arrangement within 8 days post-implantation (Fig. 8A). By day 16, these newly formed skin appendages had matured and differentiated into a more organized structure of sebaceous glands, sweat glands and hair follicles, resembling native skin. In contrast, the blank group showed only a limited number of irregular, immature, and sporadically distributed cutaneous appendages at the periphery of the extensive scar, suggesting their migration from the surrounding skin tissue towards the wound site. This was further illustrated by the elevated expression of α-SMA in the newly formed skin of the ApoEVs-AT@MNP group, which is expressed in mature fibroblasts, perivascular areas, and hair follicle sheaths (Fig. 8B). The analysis of hair follicle count and fluorescence semi-quantification further supported this assertion (Fig. 8C and D).

The thickness of type I collagen (Col 1) makes it the primary constituent of the skin, whereas type III collagen (Col 3), being smaller in size, serves as the main component of the reticular structure within the skin [32]. Among various factors influencing scar-free healing, a significant factor is the abundant presence of Col 3. This can be attributed to its small size and excellent elasticity, which enables it to easily align in an organized manner under tension during wound healing [32, 33]. Col 3 appears first at the site of injury, acting as a ‘bridge’, followed by an orderly arrangement of Col 1 fibers along a specific direction to form a stable scaffold that facilitates scarless wound healing [33–35]. To further analyze collagen composition, Col 1 and Col 3 were distinguished by IF staining. It was found that the ApoEVs-AT@MNP group had a higher abundance of the positive signals for both Col 1 and Col 3 in comparison to the blank group (Fig. 8B). There was a significant increase in the relative fluorescence density of Col 3/Col 1 in the ApoEVs-AT@MNP group (0.43) in comparison to the blank group (0.099) (Fig. 8E), contributing to scar reduction and increased skin softness. These findings provide compelling evidence that the antibacterial ApoEVs-AT@MNP can enhance the repair of skin wounds and suppress the formation of scars in a rat model.

## Discussion

The high incidence and mortality rates associated with acute and chronic wound infections impose a significant burden on global healthcare systems [1]. Despite the standardization of treatment strategies for infected wounds, the diagnosis and management of wound infections still encounter substantial challenges due to factors such as biofilm formation, delayed healing, and drug resistance [1, 2]. It is important to note that underlying systemic conditions, such as liver failure, vascular disease, diabetes mellitus, etc., can complicate the healing of infected wounds and exacerbate scar formation. Consequently, developing innovative therapeutic strategies to improve the healing process for such wounds is essential.

Given the importance of dermal adipocytes in the regeneration and development of skin, the transplantation of adipose tissue and its constituents is regarded as an advanced therapy for skin repair. Numerous earlier works have demonstrated therapeutic effects using adipose tissue, nanofat, adipose stromal cells, and additional constituents of cells to treat wounds and scars [36–39]. Nevertheless, cellular therapies face limitations due to necrosis, immune rejection, tumor risk as well as ethical concerns. Recently, exosomes, which are small extracellular vesicles, have emerged as a research hotspot in cell-free tissue engineering and have also been utilized for wound healing [40–43]. Recently, a number of studies have demonstrated that apoptosis often occurs shortly following cell transplantation [6], suggesting that apoptotic extracellular vesicles may potentially play a more significant regulatory role compared to exosomes.

ApoEVs are produced with greater efficiency from apoptotic cells, and the apoptosis process can be precisely regulated through standardized procedures [8]. In contrast to exosomes derived from living cells, vesicles obtained from adipose tissue apoptosis can be directly harvested from fatty tissue extracted during clinical liposuction surgery [11]. These vesicles are readily available, with a straightforward extraction process, and their nanoscale particle size renders them ideal for minimally invasive drug delivery, highlighting their promising potential for clinical application. Our preliminary studies have already confirmed that ApoEVs-AT can enhance skin wound regeneration. However, traditional multiple and multi-point injections pose a high risk of secondary infections in infected wounds, and the newly formed skin often lacks regularly arranged and mature skin appendages.

The microneedle array penetrates only the outermost layer of the skin, avoiding nerve endings, and creates drug delivery channels on the skin’s surface. This design targets specific depths, allowing drugs to be absorbed into the subcutaneous capillary network [44]. This facilitates painless and non-invasive drug delivery while preventing any damage to the skin. The effectiveness of microneedle mechanical stimulation in promoting the formation of hair follicles and skin appendages has been well established [19]. Recently, soluble microneedles fabricated from hydrogel have been in extensive use for tissue and organ regeneration. Upon skin penetration, the hydrogel microneedle array rapidly absorbs intercellular fluid into its mesh structure, gradually expanding the material. The hydrogel’s microchannels are created for efficient drug delivery, enabling drugs to be transported into the body through these channels via tissue fluid penetration and diffusion [44].

The goal of the current work is to fabricate an innovative transdermal drug delivery system for treating infected wounds. This system utilizes a soluble hydrogel microneedle based on HAMA as a controlled-release carrier for ApoEVs-AT, combined with an antibacterial hybrid hydrogel as a substrate consisting of PLMA and HAMA. Ultimately, a novel soluble antimicrobial microneedle patch was developed, delivering apoptotic vesicles from adipose tissue (ApoEVs-AT@MNP). This patch features soluble tips for delivering apoptotic vesicles from adipose tissue and an antibacterial substrate to inhibit bacterial growth on infected wounds.

Wounds, 2 cm in diameter, within the wound-healing model, are considered as large defects in the wound healing model and likely to result in the formation of scar [45]. In vivo implantation demonstrates the antimicrobial ApoEVs-AT@MNP not only inhibits bacterial proliferation in infected wounds but also promotes effective, rapid, and high-quality scarless wound healing. What’s more, the regenerative appearance of skin appendages, including sebaceous glands and hair follicles, is essential for the restoration of normal structure, function, and mechanical strength in injured skin. Based on our previous studies and the available literature, it is evident that the implantation of either MN or ApoEVs-AT alone, whether in the short or long term, does not lead to the formation of abundant skin appendages in the regenerated tissue [11, 12]. ApoEVs-AT@MNP utilized in this study, however, facilitated the rapid development of mature, regularly arranged hair follicles in infected wounds by day 8 post-implantation. By day 16, both mature, orderly hair follicles and visible hair were present in the wound area. Histological staining of the remaining linear scar area revealed a small number of immature skin appendages, indicating that ApoEVs-AT@MNP holds considerable promise for advancing scarless healing of infected wounds.

The research findings indicate that ApoEVs have a critical function in maintaining the population of stem cells and epithelial tissue in the skin, by activating ectomesenchymal stem cells through the Wnt/β-catenin pathway in both hair follicles and skin, thereby promoting hair growth and wound healing [8]. Concurrent research by scholars like Kim suggests that mechanical stimulation can also enhance hair growth by activating the Wnt/β-catenin signaling pathway and increasing vascular endothelial growth factor [19]. It was thus hypothesized that ApoEVs-AT@MNP could significantly enhance hair follicle regeneration and promote hair growth in full-thickness skin wounds compared to simple HAMA@MNP and ApoEVs-AT. This observed effect may be ascribed to the synergistic interaction between ApoEVs-AT and the mechanical stimulation provided by microneedles, which activate specific signaling pathways and facilitate hair regeneration through wound healing mechanisms. Further investigation is however needed to elucidate the mechanism underlying their action.

## Conclusion

In conclusion, ApoEVs-AT@MNP does not only inhibit bacterial proliferation in infected wounds but also promotes effective, rapid, and high-quality scarless healing. ApoEVs-AT@MNP facilitates the swift formation of mature, regularly arranged hair follicles in infected wounds shortly after implantation, closely mimicking natural skin regeneration. ApoEVs-AT@MNP thus represents an excellent, painless, non-invasive, and highly promising treatment option for infected wounds.

## Electronic supplementary material

Below is the link to the electronic supplementary material.

Supplementary Material 1

## Data Availability

Data is provided within the manuscript or supplementary information files.
