# Supplementary material for: An antimicrobial microneedle patch promotes functional healing of infected wounds through controlled release of adipose tissue-derived apoptotic vesicles
Source: J Nanobiotechnology. 2024 Sep 20;22:579. doi: 10.1186/s12951-024-02845-2 (PMC11414280; doi:10.1186/s12951-024-02845-2)
Supplement: Supplementary file 1 — Supplementary Material 1 [file 12951_2024_2845_MOESM1_ESM.docx]

Supplementary Information

A soluble antimicrobial microneedle transdermal delivery patch delivering apoptotic vesicles from adipose tissue to enhance healing of infected wounds

Yue Ma*, Jia Dong, Maojiao Li, Xinya Du, Zhengbin Yan,*, and Weidong Tian,*

(These authors contributed equally: Yue Ma and Jia Dong.)


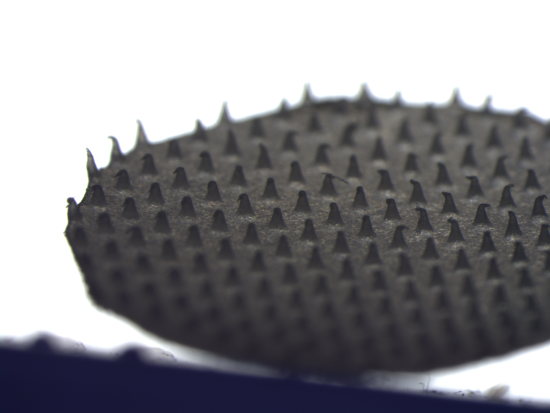


**Supplementary Fig. 1.** The general overview of the MNP axial compression experiment was observed after utilizing a universal machine.


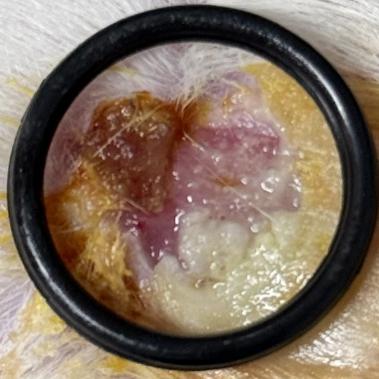


**Supplementary Fig. 2.** Part of wounds in the blank group showed aggravated infection on day 4, even after routine disinfection.

**Table S1. Oligonucleotide primer sequences.**

| Target  cDNA | Primer sequence (5 ' -- 3 ') |
| --- | --- |
| PPARγ2－F | GACCACTCCCACTCCTTTGA |
| PPARγ2－R | CAGGCTCCACTTTGATTGC |
| C/EBPα-F | AGGTTTCCTGCCTCCTTCC |
| C/EBPα-R | CCCAAGTCCCTATGTTTCCA |
| Adiponectin-F | CCCATTCGCTTTACCAAGAT |
| Adiponectin-R | GGCTGACCTTCACATCCTTC |
| FABP4-F | CAGGAAAGTCAAGAGCACCA |
| FABP4-R | TCCACCACCAGTTTATCATCC |
